# Nasopharyngeal bacterial colonization in children – study hypothesis

**DOI:** 10.1186/1471-2334-13-S1-P41

**Published:** 2013-12-16

**Authors:** Anca Streinu-Cercel, Oana Streinu-Cercel, Mihai Săndulescu, Ioana Berciu, Adrian Streinu-Cercel

**Affiliations:** 1 Carol Davila University of Medicine and Pharmacy, Bucharest, Romania; 2 National Institute for Infectious Diseases “Prof. Dr. Matei Balş”, Bucharest, Romania

## Background

With monthly reports of decreased bacterial susceptibility to antibiotics and the soaring incidence of invasive infections, it becomes increasingly important to assess bacterial colonization, as this can easily constitute a reservoir for infection. Entering the community for kindergarten and school training is an important step for children, particularly since this may be associated with a change in the microbiota.

## Methods

We aim to assess the nasal and pharyngeal flora in immune-competent children from the community, and institutionalized children with HIV infection or hematologic disorders. Bacterial colonization will be determined through the collection of nasopharyngeal swabs with subsequent culturing and species identification.

## Discussion

It has become increasingly important to describe the flora of the human body in a wide array of physiologic and pathologic conditions. Given that resident bacteria can often represent a reservoir for infection, as is the case with infectious endocarditis following dental procedures, or sepsis following digestive bacterial translocation, we aim to better describe bacterial flora by assessing nasopharyngeal colonization in healthy and immune-compromised children and comparing antibiotic sensitivity profiles.

